# Increased Incidence of Total Knee Replacement Surgery in Patients With Psoriasis: A Secondary Cohort Analysis of a Nationwide, Population-Based Health Claims Database

**DOI:** 10.3389/fmed.2021.666802

**Published:** 2021-05-11

**Authors:** Ming-Chi Lu, Kuo-Sheng Fan, Chia-Wen Hsu, Malcolm Koo, Ning-Sheng Lai

**Affiliations:** ^1^Division of Allergy, Immunology and Rheumatology, Dalin Tzu Chi Hospital, Buddhist Tzu Chi Medical Foundation, Chiayi, Taiwan; ^2^School of Medicine, Tzu Chi University, Hualien City, Taiwan; ^3^Division of Pulmonology and Critical Care, Department of Internal Medicine, Dalin Tzu Chi Hospital, Buddhist Tzu Chi Medical Foundation, Chiayi, Taiwan; ^4^Department of Medical Research, Dalin Tzu Chi Hospital, Buddhist Tzu Chi Medical Foundation, Chiayi, Taiwan; ^5^Graduate Institute of Long-term Care, Tzu Chi University of Science and Technology, Hualien City, Taiwan; ^6^Dalla Lana School of Public Health, University of Toronto, Toronto, ON, Canada

**Keywords:** psoriasis, psoriatic arthritis, total knee replacement, total hip replacement, surgery

## Abstract

Patients with rheumatic diseases, such as rheumatoid arthritis, ankylosing spondylitis, and systemic lupus erythematosus, have increased risk of receiving total knee replacement surgery or total hip replacement surgery. We speculated that psoriasis could also attack the joints of the knees and hips, leading to an increased risk of receiving total knee replacement surgery or total hip replacement surgery. The aim of this study was to investigate the risk of total knee replacement or total hip replacement surgery in patients with psoriasis using a nationwide, population-based health claims database in Taiwan. Using the Taiwan's National Health Insurance Research Database, we identified 10,819 patients with psoriasis between 2000 and 2012. A comparison cohort consisting of five patients without psoriasis for each patient with psoriasis was assembled, based on frequency matching for sex, 10-year age interval, and index year. Both groups were followed until a diagnosis of the study outcomes (total knee replacement or total hip replacement surgery) or the end of the follow-up period. Incidence rate ratios (IRRs) for the outcome variables were calculated using multiple Poisson regression models. Female patients with psoriasis exhibited a significantly higher incidence of receiving total knee replacement surgery [adjusted IRR = 1.44, *p* = 0.014)]. Analyses stratified by age groups showed that the risk of receiving total knee replacement surgery was significantly higher older (adjusted IRR = 1.31, *p* = 0.047) patients with psoriasis. There were no significant differences in the risk of receiving total hip replacement surgery in patients with psoriasis compared with controls, either with or without stratification by sex or age groups. In conclusion, patients with psoriasis were associated with an increased risk of receiving total knee. Clinicians should be vigilant in assessing the presence of arthritis in these patients, and initiate strategies to delay or prevent the need for joint replacement.

## Introduction

Psoriasis is a common, chronic, non-communicable, inflammatory skin disease characterized by erythematous, scaly patches, or plaques on the skin (1). The worldwide prevalence of psoriasis was estimated to vary from 0.14 to 1.99% (2). The debilitating and highly visible skin symptoms of psoriasis can severely impact on patients' quality of life (3). Up to one-third of patients with psoriasis could develop psoriatic arthritis, which is a chronic and potentially severe condition. It can cause joint damage leading to deformity, and may require surgery to alleviate pain and restore function (4). Our previous studies have shown that patients with rheumatic diseases, such as rheumatoid arthritis, ankylosing spondylitis, and systemic lupus erythematosus, have increased risk of receiving total knee replacement (TKR) surgery or total hip replacement (THR) surgery (5–7). We speculated that psoriasis could also attack the joints of the knees and hips resulting in their destruction, which then lead to an increased risk of receiving TKR and THR. Therefore, the aim of this study was to investigate the incidence of THR and TKR in patients with psoriasis using a nationwide, population-based health claims database in Taiwan.

## Materials and Methods

### Study Design and Data Source

This study is a secondary analysis of a nationwide, population-based, retrospective cohort based on the data available from the National Health Insurance Research Database (NHIRD). The study protocol was approved by the institutional review board of the Dalin Tzu Chi Hospital, Buddhist Tzu Chi Medical Foundation, Taiwan (No. B10104020). The requirement for obtaining informed consent from patients was waived because the data file contained only deidentified secondary data.

### Identification of the Psoriasis Cohort and a Comparison Cohort

Patients in the psoriasis cohort were identified based on the International Classification of Diseases, 9^th^ Revision, Clinical Modification (ICD-9-CM) codes 696.0, 696.1, and 696.8, using the ambulatory care expenditures by visits datafile of the Longitudinal Health Insurance Database (LHID 2000) from January 1, 2000 to December 31, 2012. The comparison cohort was assembled from the patients in LHID 2000. Five patients without psoriasis were selected, based on frequency matching for 10-year age interval and index year, for each patient with psoriasis.

In both cohorts, patients with systemic lupus erythematosus (ICD-9-CM code 710.0), rheumatoid arthritis (ICD-9-CM code 714.0), ankylosing spondylitis (ICD-9-CM code 720.0), juvenile rheumatoid arthritis (ICD-9-CM code 714.3X), and acquired immune deficiency syndrome (ICD-9-CM code: 042) were excluded. In addition, patients with fracture of the lower limb (ICD-9-CM codes 820–829), obesity (ICD-9-CM code 278.0x), osteoarthritis (ICD-9-CM code 715.xx), and avascular necrosis (ICD-9-CM code 733.4x) were identified and these conditions were adjusted as potential confounders. Patients younger than 20 years or older than 80 years of age were also excluded.

### Identification of Total Knee Replacement Surgery and Total Hip Replacement Surgery

We followed all patients until the occurrence of our study events or the end of the follow-up period, separately for the TKR and THR outcome variables. TKR and THR were defined in this study using inpatient ICD-9-CM procedure codes 81.54 and 81.51, respectively. For the analysis of the risk of TKR, patients in the psoriasis and comparison cohorts receiving TKR before the index date were excluded. For the analysis of the risk of THR, those receiving THR before the index date were excluded.

### Statistical Analysis

We compared the basic characteristics between the psoriasis cohort and the comparison cohort using *t*-test or Chi-square test, as appropriate. For the psoriasis cohort and the comparison cohort, the incidence rate per 1,000 person-years was separately calculated for TKR and THR. Incidence rate ratios (IRRs) for the outcome variables were calculated using Poisson regression models (generalized linear models with a Poisson log-linear link function and person-years as the offset variable), with and without adjusting for potential confounding factors, including fractures of the lower limb, obesity, osteoarthritis, osteonecrosis, age, sex, socioeconomic status, and geographic region. In addition, subgroup analyses were conducted with stratification by age groups (20–44, 45–64, and 65–80 years). All statistical analyses were performed using IBM SPSS Statistics software package, version 24.0 (IBM Corp, Armonk, NY, USA). A *p*-value of < 0.05 was considered significant.

## Results

There were no significant differences between the psoriasis cohort and the comparison cohort in age and sex. Patients with psoriasis showed a higher socioeconomic status and different geographic distribution compared with the comparison cohort. Patients with psoriasis had a significantly higher proportion of obesity (*p* = 0.004), osteoarthritis (*p* < 0.001), and avascular necrosis (*p* < 0.001), but a significantly lower proportion of fractures of the lower limb (*p* = 0.046) compared to those in the comparison cohort (Table 1).

**Table 1:** Basic characteristics of the psoriasis cohort and comparison cohort (*N* = 64,914).

**Variable**	***N*** **(%)**				***P***
	**Psoriasis cohort 10,819 (16.7)**		**Comparison cohort 54,095 (83.3)**		
Sex					>0.999
male	6,197	(57.3)	30,985	(57.3)	
female	4,622	(42.7)	23,110	(42.7)	
Age group (years)					>0.999
20–39	4,182	(38.7)	20,910	(38.7)	
40–59	4,045	(37.3)	20,225	(37.3)	
60–80	2,592	(24.0)	12,960	(24.0)	
Mean age (standard deviation), years	46.3	(16.6)	46.3	(16.6)	0.988
Median age (interquartile range), years	45	(32–59)	45	(32–59)	
Obesity	38	(0.4)	112	(0.2)	0.004
Fracture of the lower limb	38	(0.4)	268	(0.5)	0.046
Osteoarthritis	424	(3.9)	1,157	(2.1)	<0.001
Avascular necrosis	20	(0.2)	32	(0.1)	<0.001
Socioeconomic status (*n* = 64,879)					<0.001
low	5,483	(50.7)	30,115	(55.8)	
middle	2,887	(26.7)	14,019	(25.9)	
high	2,449	(22.6)	9,886	(18.3)	
Geographic region (*n* = 63,237)					<0.001
Northern	5,864	(55.6)	32,748	(62.2)	
Central	1,539	(14.6)	8,629	(16.4)	
Southern	2,943	(27.9)	10,260	(19.5)	
Eastern	202	(1.9)	1,052	(2.0)	

*Socioeconomic status was estimated by insurance premiums based on salary. Low: ≤ 19,000 New Taiwan dollars (NT$); middle: 19,001–24,000; and high: > 24,000*.

Table 2 shows the incidence rates, IRRs, and adjusted IRRs of TKR for the psoriasis cohort and the comparison cohort. Patients with psoriasis showed a significantly higher incidence of receiving TKR compared with the comparison cohort (adjusted IRR 1.38, *p* = 0.007). In addition, with subgroup analyses stratified by sex, female patients, but not male patients with psoriasis had a significantly higher incidence of receiving TKR (adjusted IRR 1.44, *p* = 0.014) compared with the comparison cohort. Furthermore, in analyses stratified by the three age groups, only patients with psoriasis showed an increased incidence in TKR in the 60–80 year group (adjusted IRR 1.31, *p* = 0.047).

**Table 2:** The incidence rate and incidence risk ratio of total knee replacement in the psoriasis cohort and comparison cohort (*N* = 64,672).

**Disorder (ICD-9-CM)**		**Psoriasis cohort (*****n*** **=** **10,764)**			**Comparison cohort (*****n*** **=** **53,908)**			**IRR (95% CI)**	aIRR^*^ (95% CI)
		**No. of patient**	**Person-years**	**IR**	**No. of patient**	**Person-years**	**IR**	***P***	***P***
TKR (815.4)	Overall	96	68,510	1.40	297	325,346	0.91	1.54 (1.22–1.93) <0.001	1.38 (1.09–1.75) 0.007
	Sex								
	male	33	39,013	0.85	111	185,879	0.60	1.42 (0.96–2.09) 0.079	1.29 (0.87–1.92) 0.209
	female	63	29,497	2.14	186	139,467	1.33	1.60 (1.20–2.13) 0.001	1.44 (1.08–1.93) 0.014
	Age group (years)								
	20–39	1	27,750	0.04	3	132,933	0.02	1.60 (0.17–15.35) 0.685	2.84 (0.26–31.36) 0.394
	40–59	21	25,817	0.81	56	125,788	0.45	1.83 (1.11–3.02) 0.018	1.68 (1.00–2.81) 0.051
	60–80	74	14,943	4.95	238	66,625	3.57	1.39 (1.07–1.80) 0.014	1.31 (1.00–1.71) 0.047

*aIRR, adjusted incidence rate ratio; CI, confidence interval; ICD-9-CM, International Classification of Diseases, Ninth revision, clinical modification; IR, incidence rate per 1,000 person-years; IRR, incidence rate ratio; TKR, total knee replacement.*

* *Adjusted for age, sex, socioeconomic status, geographic region, obesity, fracture of the lower limb, osteoarthritis, and avascular necrosis*.

Table 3 shows the incidence rates, IRRs, and adjusted IRRs of THR for the psoriasis cohort and the comparison cohort. The overall adjusted IRR for receiving THR in patients with psoriasis was not significantly different between the two cohorts. Similarly, subgroup analyses stratified by either the three age groups or sex were not significantly different between the two cohorts in the risk of THR.

**Table 3:** The incidence rate and incidence risk ratio of total hip replacement in the psoriasis cohort and comparison cohort (*N* = 64,781).

**Disorder (ICD-9-CM)**		**Psoriasis cohort (*****n*** **=** **10,782)**			**Comparison cohort (*****n*** **=** **53,999)**			**IRR (95% CI)**	aIRR^*^ (95% CI)
		**No. of patient**	**Person-years**	**IR**	**No. of patient**	**Person-years**	**IR**	***P***	***P***
THR (815.1)	Overall	37	68,548	0.54	137	325,555	0.42	1.28 (0.89–1.84) 0.179	1.27 (0.88–1.84) 0.204
	Sex								
	male	27	38,931	0.69	89	185,755	0.48	1.45 (0.94–2.23) 0.092	1.40 (0.90–2.19) 0.137
	female	10	29,617	0.34	48	139,800	0.34	0.98 (0.50–1.94) 0.962	1.09 (0.55–2.19) 0.803
	Age group (years)								
	20–39	5	27,716	0.18	21	132,919	0.16	1.14 (0.43–3.03) 0.790	1.18 (0.42–3.32) 0.757
	40–59	19	25,725	0.74	61	125,611	0.49	1.52 (0.91–2.54) 0.111	1.55 (0.91–2.65) 0.106
	60–80	13	15,107	0.86	55	67,025	0.82	1.05 (0.57–1.92) 0.878	0.97 (0.53–1.79) 0.923

*aIRR, adjusted incidence rate ratio; CI, confidence interval; ICD-9-CM, international classification of diseases, Ninth revision, clinical modification; IR, incidence rate per 1,000 person-years; IRR, incidence rate ratio; THR, total hip replacement.*

* *Adjusted for age, sex, socioeconomic status, geographic region, obesity, fracture of the lower limb, osteoarthritis, and avascular necrosis*.

## Discussion

This secondary cohort analysis of a nationwide, population-based health claim database showed that patients with psoriasis, especially female and those middle-aged or older, had a significantly higher risk of receiving TKR. On the other hand, male and those middle-aged patients with psoriasis had a significantly higher risk of receiving THR. It is known that women are more likely to receiving TKR in the general population (8). Knee joint inflammation has been demonstrated in patients with psoriasis (9), and our finding was consistent with their findings. Although the presence of detection bias was possible as a result of increased medical utilization in our patients, we believe its effects on my results are minimal. First, patients generally would only consider TKR and THR surgery when they have severe joint damage over the hip or knee joint, which is accompanied with obvious pain and disability. Second, prior to TKR and THR surgery, an approval from the Taiwan National Health Insurance Administration, which requires a review of X-ray films of the target joint and clinical data by an orthopedic specialist (5). As these factors are not related to increased medical surveillance, our results should not be affected by detection bias.

Prior to this study, we anticipated that some patients with psoriasis would develop psoriatic arthritis, causing chronic and destructive inflammation of joints and thereby leading to an increased risk of receiving TKR or THR. However, we found that only four (4.2%) patients with psoriasis were diagnosed with psoriatic arthritis before TKR and only three (8.1%) patients were diagnosed with psoriatic arthritis before THR in our study. The number of cases is too few to analyze. The percentage of psoriatic arthritis in patients with psoriasis has been reported to be 20–30% (4). Nevertheless, the percentage of psoriatic arthritis in patients with psoriasis was found to be only 8.2% based on a study using the NHIRD in Taiwan (10). A study based on 34 dermatology centers in seven European and North American countries found that approximately a third of patients with psoriasis seen in dermatology centers had psoriatic arthritis as assessed by rheumatologists. Among the patients with psoriatic arthritis, 41% had not been previously given the diagnosis (11). A meta-analysis based on seven epidemiological studies and five studies on psoriatic arthritis screening questionnaires also supported that there is a high prevalence of undiagnosed psoriatic arthritis in patients with psoriasis (12). In a cross-sectional clinical survey of 414 patients, the percentage of psoriatic arthritis patients with psoriasis was found to be 30.6% (13). Therefore, we believed that psoriatic arthritis was underdiagnosed in the NHIRD. In addition, there are many therapeutic agents available for treating psoriatic arthritis (14). Early treatment and tight control of inflammation in early psoriatic arthritis can clearly improve patients' outcome (15). Therefore, clinicians need to be vigilant regarding the possible psoriatic arthritis in patients with psoriasis.

There are some limitations in this study. First, the identification of psoriasis, psoriatic arthritis, THR, and TKR were based on ICD-9-CM codes. Nevertheless, the National Health Insurance Administration routinely performs audits on random samples of medical claims to ensure their accuracy. Second, the severity of psoriasis could not be obtained because the NHIRD data did not include clinical assessments. Despite these limitations, the strengths of this study included the large sample size, a population-based cohort study design, and a long follow-up period.

In conclusion, this study showed that female and older patients with psoriasis had a higher risk of receiving TKR. Patients with psoriasis did not show an increased risk for receiving THR. Clinicians should be vigilant in assessing the presence of arthritis in patients with psoriasis, and initiate strategies to delay or prevent the need for joint replacement in these patients.

## Data Availability Statement

The data analyzed in this study is subject to the following licenses/restrictions: Due to legal restrictions imposed by the government of Taiwan in relation to the “Personal Information Protection Act,” data cannot be made publicly available. Requests for data can be sent as a formal application to the Health and Welfare Data Science Center, Department of Statistics, Ministry of Health and Welfare, Taiwan. Requests to access these datasets should be directed to http://dep.mohw.gov.tw/DOS/np-2497-113.html.

## Ethics Statement

The studies involving human participants were reviewed and approved by the institutional review board of the Dalin Tzu Chi Hospital, Buddhist Tzu Chi Medical Foundation, Taiwan (No. B10104020). Written informed consent for participation was not required for this study in accordance with the national legislation and the institutional requirements.

## Author's Note

This study is based in part on data from the National Health Insurance Research Database provided by the National Health Insurance Administration, Ministry of Health and Welfare and managed by the National Health Research Institutes, Taiwan. The interpretation and conclusions contained herein do not represent those of the National Health Insurance Administration, Ministry of Health and Welfare or the National Health Research Institutes, Taiwan.

## Author Contributions

M-CL, K-SF, and N-SL conceptualized the idea of the manuscript. C-WH conducted statistical analysis of data. M-CL and MK wrote the manuscript. All authors have read and approved the contents of the final manuscript.

## Conflict of Interest

The authors declare that the research was conducted in the absence of any commercial or financial relationships that could be construed as a potential conflict of interest.
